# Biopsy-Proven T-Cell Mediated Rejection After Belatacept Rescue Conversion: A Multicenter Retrospective Study

**DOI:** 10.3389/ti.2024.13544

**Published:** 2024-12-06

**Authors:** Dominique Bertrand, Nathalie Chavarot, Jérôme Olagne, Clarisse Greze, Philippe Gatault, Clément Danthu, Charlotte Colosio, Maïté Jaureguy, Agnès Duveau, Nicolas Bouvier, Yannick Le Meur, Léonard Golbin, Eric Thervet, Antoine Thierry, Arnaud François, Charlotte Laurent, Mathilde Lemoine, Dany Anglicheau, Dominique Guerrot

**Affiliations:** ^1^ Department of Nephrology, Kidney Transplantation and Hemodialysis, Rouen University Hospital, Rouen, France; ^2^ Department of Nephrology and Kidney Transplantation, Necker-Enfants Malades Hospital, Assistance Publique-Hôpitaux de Paris, Paris, France; ^3^ Department of Nephrology, Kidney Transplantation and Hemodialysis, Strasbourg University Hospital, Strasbourg, France; ^4^ Department of Nephrology, Kidney Transplantation and Hemodialysis, Clermont-Ferrand University Hospital, Clermont-Ferrand, France; ^5^ Department of Nephrology, Kidney Transplantation and Hemodialysis, Tours University Hospital, Tours, France; ^6^ Department of Nephrology, Kidney Transplantation and Hemodialysis, Limoges University Hospital, Limoges, France; ^7^ Department of Nephrology, Kidney Transplantation and Hemodialysis, Reims University Hospital, Reims, France; ^8^ Department of Nephrology, Kidney Transplantation and Hemodialysis, Amiens University Hospital, Amiens, France; ^9^ Department of Nephrology, Kidney Transplantation and Hemodialysis, Angers University Hospital, Angers, France; ^10^ Department of Nephrology, Kidney Transplantation and Hemodialysis, Caen University Hospital, Caen, France; ^11^ Department of Nephrology, Kidney Transplantation and Hemodialysis, Brest University Hospital, Brest, France; ^12^ Department of Nephrology, Kidney Transplantation and Hemodialysis, Rennes University Hospital, Rennes, France; ^13^ Department of Nephrology and Dialysis, Hôpital Européen Georges Pompidou, Assistance Publique-Hôpitaux de Paris, Paris, France; ^14^ Department of Nephrology, Kidney Transplantation and Hemodialysis, Poitiers University Hospital, Poitiers, France; ^15^ Department of Pathology, Rouen University Hospital, Rouen, France

**Keywords:** transplantation, kidney, belatacept, rejection, CNI toxicity

## Abstract

After kidney transplantation, conversion to belatacept is a promising alternative in patients with poor graft function or intolerance to calcineurin inhibitors. The risk of acute rejection has not been well described under these conditions. Here we present a retrospective multicenter study investigating the occurrence of acute rejection after conversion in 901 patients (2011–2021). The incidence of cellular and humoral rejection was 5.2% and 0.9%, respectively. T-cell mediated rejection (TCMR) occurred after a median of 2.6 months after conversion. Out of 47 patients with TCMR, death-censored graft survival was 70.1%, 55.1% and 50.8% at 1 year, 3 years and 5 years post-rejection, respectively. Eight patients died after rejection, mainly from infectious diseases. We compared these 47 patients with a cohort of kidney transplant recipients who were converted to belatacept between 2011 and 2017 and did not develop rejection (n = 238). In multivariate analysis, shorter time between KT and conversion, and the absence of anti-thymocyte globulin induction after KT were associated with the occurrence of TCMR after belatacept conversion. The occurrence of rejection after conversion to belatacept appeared to be less frequent than with *de novo* use. Nevertheless, the risk of graft loss could be significant in patients with already low renal function.

## GraphicalAbstract

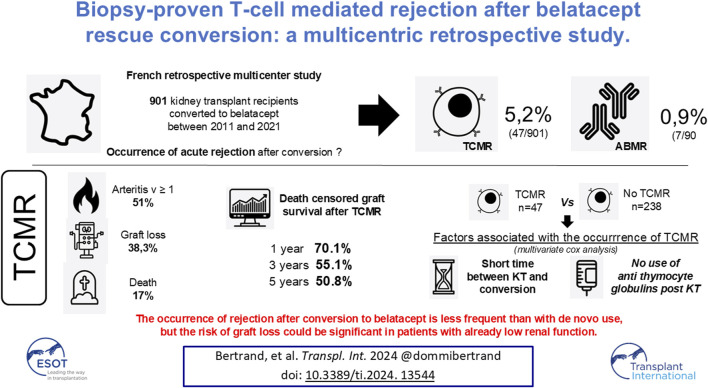

## Introduction

Belatacept is an immunosuppressive drug that blocks the costimulation pathway, preventing T cell activation. With this different mechanism of action, belatacept represents an alternative to calcineurin inhibitors (CNIs) after kidney transplantation and could have major advantages. When used as a *de novo* therapy post-transplantation, belatacept improved long-term graft function, graft survival and patient survival in the BENEFIT study [1]. Moreover its metabolic profile is better than CNIs [2] and the rate of *de novo* DSA is lower [3]. When used as a conversion strategy, the randomized study by Budde et al. [4] also reported benefits for graft function and for the rate of *de novo* DSA, in stable KTRs. Furthermore, there is growing evidence that CNIs to belatacept conversion is a valuable option as rescue therapy in patients with poor graft function [5]. A major pitfall and obstacle to more widespread use of belatacept in *de novo* KTRs is the particularly high rate of TCMR (T cell-mediated rejection) occurring in up to 24% of patients in the BENEFIT [1] PRINCEPS study. The rejection rate seems to be lower in conversion strategies ranging between 5.3% and 11.4 % according to various studies [4, 6–10] and was not significantly different between the belatacept and CNI arms in our retrospective study [5]. However some of these rejections are steroid-resistant TCMRs [11, 12] and could lead to accelerated graft loss. Unfortunately, there are no reports of risk factors or biomarkers associated with the occurrence of rejection in this context.

We designed a multicenter retrospective study in which we included all patients who were converted to belatacept over a 10-year period who presented with biopsy-proven rejection. The aims of the present study were to report the incidence of both TCMR and ABMR (antibody-mediated rejection) after conversion to belatacept in a rescue strategy, to depict the evolution of these patients and to identify factors associated with the occurrence of TCMR after conversion.

## Materials and Methods

### Study Design: Flow Charts (Figure 1) and Patients

We conducted a retrospective study, between 2011 and 2021, in which all the kidney transplant recipients (KTRs) from the Spiesser group (13 French KT centers) who presented a biopsy-proven rejection after belatacept conversion were included (all were for cause biopsies). Conversion was performed for poor graft function and/or intolerance to calcineurin inhibitors. Histological features of the kidney allograft biopsies were scored according to the Banff classification [13]. During this period a total of 901 KTRs were converted to belatacept.

**FIGURE 1:**
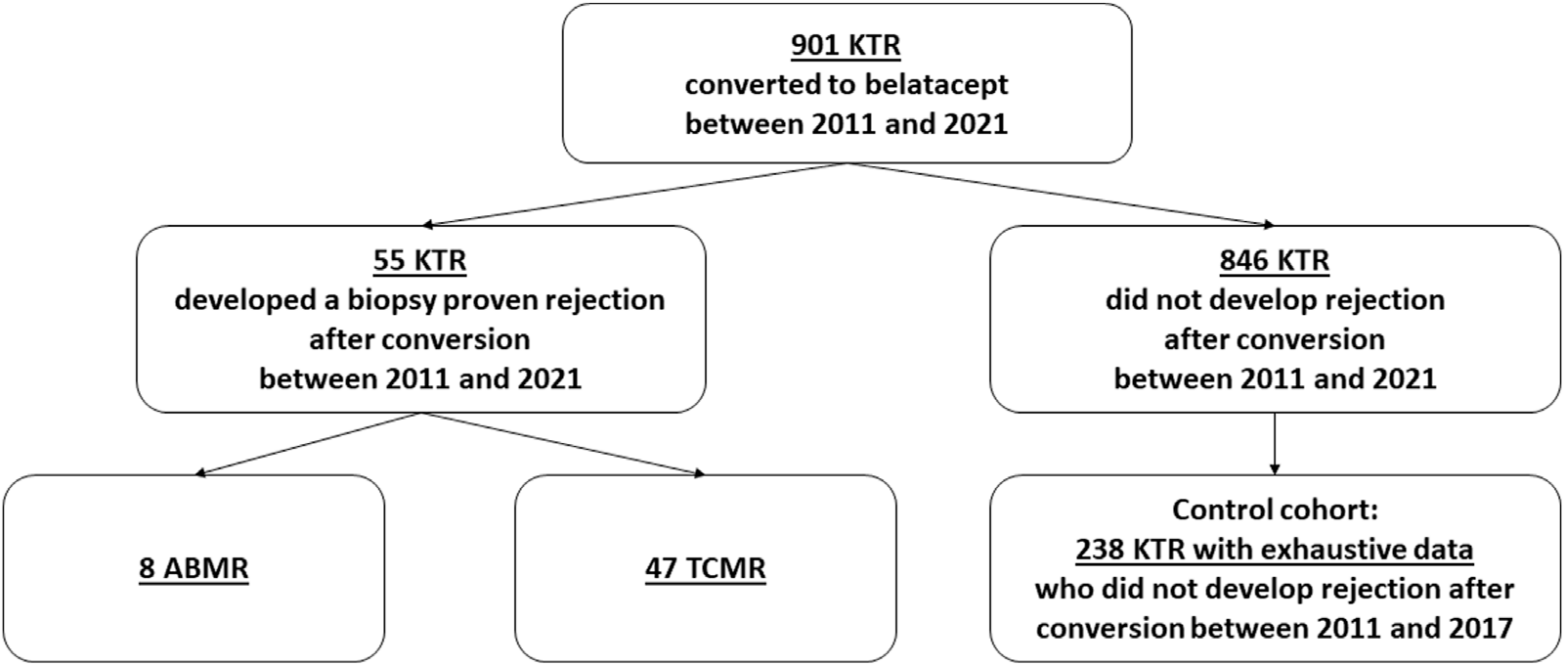
Flow chart. KTRs, kidney transplant recipients; ABMR, antibody-mediated rejection; TCMT, T cell-mediated rejection.

In accordance with French law (loi Jardé), because this was an anonymous retrospective study, institutional review board approval was not required. The clinical and research activities being reported are consistent with the Principles of the Declaration of Istanbul as outlined in the “Declaration of Istanbul on Organ Trafficking and Transplant Tourism.”

### Treatment

The CNI to belatacept conversion group consisted of 5 mg/kg of belatacept administered intravenously on days 1, 15, 29, 43, 57 and then 28 days thereafter [14]. CNIs were tapered as follows: 100% on day 1, 50% on day 2, 25% on day 15, and 0 from day 29 onwards. Other immunosuppressive medications, including corticosteroids, were maintained at existing doses unless modification was necessary. All patients were EBV seropositive before the conversion. Beginning in January 2019, all patients converted to belatacept received pneumocystis prophylaxis. Patients who received belatacept as *de novo* therapy were excluded.

### Primary Outcome: Kidney Transplant Recipients With Rejection After Conversion

The primary endpoint was the rate of both TCMR and ABMR after conversion to belatacept. We excluded patients who experienced ABMR due to the small number of them and focused on TCMR in order to determine factors associated with graft loss. We defined graft failure as a return to chronic dialysis. We evaluated kidney graft function using eGFR (MDRD) [15].

### Secondary Outcomes: Rate of Opportunistic Infection (OPI) and Factors Associated With the Occurrence of TCMR After Conversion to Belatacept

During the study period, 901 KTRs were converted to belatacept: 55 KTRs developed a biopsy-proven rejection while the 846 others did not. Among the 846 patients without rejection, 238 KTRs [6], well-phenotyped with exhaustive data, converted between 2011 and 2017, were analyzed and compared to the “rejection cohort” to identify the incidence of OPIs and factors associated with the occurrence of TCMR after conversion. Moreover, there was no difference between 2011–2017 and 2018–2021 regarding the protocol of conversion used (dose of belatacept, timing of discontinuation of CNIs).

All OPIs occurring under belatacept therapy were recorded in our medical charts. Infection was defined by a specific clinical/biological/radiological presentation and the finding of a causal infectious agent (bacterial, viral, fungal or parasitic). The nature of the infection (microbiological causative agents) and the localization of the infection were recorded. The lymphocyte count was reviewed at the time of the switch for all patients. We considered OPIs as described by Fishman in 2007 [16]: pneumocystis pneumonia; infection with herpes viruses (herpes simplex virus, varicella-zoster virus, Cytomegalovirus, Epstein-Barr virus and others); infection with *listeria*, *nocardia*, toxoplasma, strongyloides, leishmania, Trypanosoma cruzi; polyomavirus BK nephropathy; Cryptococcus neoformans infection; *Mycobacterium tuberculosis* or atypical mycobacteria infection; infection with aspergillus, atypical molds, mucor species; infection with JC polyomavirus [progressive multifocal leukoencephalopathy (PML)].

### Statistical Methods

Quantitative data were presented as mean (SD), or median (interquartile range IQR) when data were not normally distributed. Qualitative data were presented as percentages. Non-parametric Wilcoxon (quantitative data) and Mann-Whitney (qualitative data) tests were used to compare baseline characteristics. Univariate and multivariable Cox regression analyses were performed to determine independent covariates associated with the occurrence of TCMR: age, gender, time between KT and conversion, the use of anti-thymocyte globulin induction post KT, extended criteria donor, eGFR at conversion, lymphocyte counts at conversion, tacrolimus before conversion, the time between conversion and CNI discontinuation, MMF at time of TCMR or month 3 post-conversion in KTRs without TCMR, MMF dose, steroids at the time of TCMR or month 3 post-conversion in KTRs without TCMR, and steroid dose. All factors with *P* < 0.1 in the univariate analysis were included in the multivariate model. *P* < 0.05 was considered statistically significant in the multivariate model. Results were presented as a hazard ratio (HR) and a 95% confidence interval (CI). For Cox models, we tested the validity of the proportional hazards assumption using the Scaled Schoenfeld vs. time graph for each variable. There was no violation of the proportional hazards assumption. We tested the interaction between the variables in the final model using a parameter covariance matrix to show how much each parameter was correlated with each other. All analyses were performed using STATVIEW version 5.0 (SAS Institute, Cary, NC, United States) and GraphPad Prism version 8.0 software (GraphPad Software, San Diego, CA).

## Results

### Incidence of Rejection After Belatacept Conversion During the Period 2011–2021

Between 2011 and 2021, 901 patients were converted from CNIs to belatacept after kidney transplantation. Of these 55 (6.1%) patients, who were converted after a median time of 3.6 months (IQR: 1.1–9.5) post-transplant developed a biopsy-proven acute rejection after a median time of 2.6 months post-conversion (IQR: 2.1–4.1 months). Of these, 47 (85.4%) developed TCMR and 8 (14.6%) ABMR. The incidence of TCMR and ABMR during this period was 5.2% and 0.9% respectively. None of the patients had a rejection prior to conversion.

We noted a substantial decrease in the rejection rate (TCMR) over time: 2011–2017: 18/256 = 7% and 2017–2021: 29/645 = 4.5%.

### Kidney Transplant Recipients With TCMR: Clinical, Biological and Histological Characteristics at the Time of Diagnosis

Regarding TCMR, according to the Banff classification [13], we reported borderline lesions in 5 cases (10.7%), 9 grade IA (19.1%), 9 grade IB (19.1%), 7-grade IIA (14.9%), 11 grade IIB (23.4%) and 6 grade III (12.8%) TCMR. Biopsies of TCMR revealed v lesions in 24/47 cases (51%).

The general characteristics of KTRs with TCMR are reported in Table 1. Kidney transplant recipients with biopsy-proven rejection presented at the time of diagnosis with a decrease in eGFR from a median of 25.5 mL/min/1.73 m^2^ (IQR: 14.5–32.1) at the time of conversion to 16.2 mL/min/1.73 m^2^ (IQR: 9.9–24.6) at the time of rejection. Five KTRs (10.6%) required dialysis at the time of rejection.

**TABLE 1:** Clinical and biological characteristics of patients with and without T cell-mediated rejection (TCMR) (historical cohort).

	KTRs with TCMR n = 47	KTRs without TCMR (historical cohort) n = 238	*p*
Sex M/F n (%)	36 (76.6)/11 (23,4)	144 (60.5)/94 (39,5)	0.04
Age at conversion (years), mean ± SD	56.9 ± 13.9	56.2 ± 14.8	0.94
Mean time between KT and conversion (months) median time (IQR) Conversion before 6 months post KT n(%)	3.6 (1.0–9.1) 28 (59.6)	13.2 (4.1–51.3) 82 (34.5)	<0.0001 0.001
Use of anti thymocyte globulins post KT	10 (21.3)	89 (37.4)	0.03
ECD n(%)	34 (72.3)	136 (57.1)	0.05
eGFR at conversion (MDRD. mL/min/1.73 m^2^). mean ± SD	27.0 ± 17.4	27.3 ± 15.3	0.66
Lymphocytes count at conversion (/mm^3^) mean ± SD	1,170 ± 613	1,070 ± 668	0.19
Treatment prior to conversion n (%) Tacrolimus MMF Steroids	40 (85.1) 44 (93.6) 37 (78.7)	167 (70.2) 208 (87.4) 205 (86.1)	0.04 0.66 0.22 0.19
Mean time between conversion and CNI discontinuation (months) Median time (IQR)	0.9 (0.5–1.1)	0.9 (0.88–1.0)	0.71
Treatment at time of TCMR or at month 3 n(%) MMF Median dose (IQR) Steroids Median dose (IQR)	42 (89.4) 1,250 (1,000–2,000) 39 (82.9) 10 (7.5–10)	220 (92.4) 1,000 (1,000–1,500) 205 (86.1) 10 (5–10)	0.48 0.47 0.57 0.19

M/F, male subjects/female subjects; KT, kidney transplantation; eGFR, estimated glomerular filtration rate; CNIs, calcineurin inhibitors; MMF, mycophenolate mofetil.

### Kidney Transplant Recipients With TCMR: Evolution After Treatment

All KTRs were treated with high doses of steroids after the diagnosis of TCMR: 43 (91.5%) with intravenous infusion and 4 (8.5%) with oral treatment. Moreover, of the 47 KTRs, 7 (14.9%) were treated with anti-thymocyte globulin. Twelve patients (25.5%) were resistant to treatment. After treatment, 33 patients (70.2%) recovered an eGFR at least equivalent to that at the time of the conversion, from 17.2 mL/min/1.73 m^2^ (IQR: 12.6–28.9) at the time of rejection to 35.1 mL/min/1.73 m^2^ (IQR: 24.3–43.2) after treatment. After treatment, belatacept was discontinued and CNIs were resumed in 18 KTRs (38.3%). Belatacept was continued in the remaining 29 KTRs (61.7%).

After TCMR, 8 deaths were reported within 13.3 months (IQR: 9.1–34.4) after rejection, 7 of which were of infectious origin: 3 deaths from invasive aspergillosis, 2 from bacterial pneumonia, one from uncontrolled bacterial osteitis and one from influenza virus. After TCMR, 18 graft losses were reported after a median time of 7.1 months (IQR: 1.3–15.9) after rejection. Death-censored graft survival was 70.1%, 55.1% and 50.8% at 1 year, 3 years and 5 years post rejection, respectively.

In KTRs without graft loss, median eGFR increased from 18.9 mL/min/1.73 m^2^ (IQR: 14.1–29.7) at the time of rejection to 35.1 mL/min/1.73 m^2^ (IQR: 28.9–45.7) after treatment and to 34.4 mL/min/1.73 m^2^ (IQR: 24.3–41.4) 1-year post rejection.

### Factors Associated With Graft Loss After TCMR

Characteristics of KTRs with TCMR and graft loss (n = 18) compared to those without graft loss (n = 29) are reported in Table 2. The discontinuation of belatacept after rejection and the eGFR at the time of rejection were significantly associated with graft loss after TCMR.

**TABLE 2:** Clinical, biological and histological characteristics of patients with T cell-mediated rejection (TCMR) with or without graft loss.

	TCMR and graft loss n = 18	TCMR without graft loss n = 29	*p*
Sex M/F n (%)	12(66.7)/6 (33.3)	24 (82.8)/5 (17.2)	0.20
Age at conversion (years), mean ± SD	55.5 ± 14.0	59.1 ± 13.7	0.32
Mean time between KT and conversion (months) median time	3.3	4.3	0.70
Interval between rejection and conversion (months) median time	2.3	2.6	0.11
ECD n(%)	15 (83.3)	19 (65.5)	0.18
eGFR at the time of conversion (MDRD. mL/min/1.73 m^2^) mean ± SD	23.9 ± 21.2	28.9 ± 14.7	0.07
eGFR at the time of rejection (MDRD. mL/min/1.73 m^2^) mean ± SD	10.7 ± 6.5	23.7 ± 13.6	0.0001
Discontinuation of belatacept after TCMR	12 (66.6)	6 (20.7)	0.002
Banff lesion g+ptc median (IQR)	2.2 ± 1.5	1.4 ± 1.1	0.08
Banff lesion i+t median (IQR)	4.2 ± 1.7	3.8 ± 1.6	0.37
Banff lesion v median (IQR)	1.1 ± 1.1	1.2 ± 1.1	0.63
Banff lesion ci+ct median (IQR)	1.8 ± 1.9	2.6 ± 1.8	0.23
Banff lesion cv+ah median (IQR)	2.3 ± 1.5	2.8 ± 1.7	0.37

M/F, male subjects/female subjects; KT, kidney transplantation; eGFR, estimated glomerular filtration rate; CNIs, calcineurin inhibitors; MMF, mycophenolate mofetil; F, female; ECD, extended criteria donor; eGFR, estimated glomerular filtration rate; Banff scores: ah arteriolar hyalinosis, ci interstitial fibrosis, ct tubular atrophy, cv vascular fibrous intimal thickening; g, glomerulitis score; i, interstitial inflammation; ptc, peritubular capillaritis score; v, arteritis score.

### Factors Associated With the Occurrence of TCMR After Conversion to Belatacept

We compared the 47 KTRs with TCMR during the period 2011–2021 with a subset of the cohort converted to belatacept between 2011 and 2017 who did not develop rejection (n = 238) [6]. General patient characteristics are reported in Table 1.

Univariate and multivariate Cox analyses to determine factors associated with the occurrence of TCMR after belatacept conversion are reported in Table 3. In multivariate analysis, the time between KT and conversion, and the absence of anti-thymocyte globulin treatment as an induction after KT were associated with the occurrence of TCMR after belatacept conversion.

**TABLE 3:** Univariate and multivariate Cox analyses for determining factors associated with the occurrence of T cell-mediated rejection (TCMR) after belatacept conversion.

	Univariate analysis			Multivariate analysis		
	HR	IC 95%	*p*	HR	IC 95%	*p*
Sex F	*0.48*	*0.25–0.95*	*0.03*	*0.68*	*0.32–1.41*	*0.29*
Age at conversion	1.01	0.98–1.02	0.69			
Time between KT and conversion	***0.97***	***0.95–0.99***	***0.002***	***0.97***	***0.94–0.99***	***0.01***
No use of anti thymocyte globulins post KT	***2.06***	***1.03–4.15***	***0.04***	***2.51***	***1.14–5.56***	***0.02***
Non ECD	*0.53*	*0.28–1.01*	*0.05*	*1.01*	*0.50–2.02*	*0.99*
eGFR at the time of conversion (MDRD. mL/min/1.73 m^2^)	0.99	0.98–1.02	0.82			
Lymphocyte count at conversion	1.00	1–1.01	0.34			
No tacrolimus before conversion	*0.43*	*0.19–0.96*	*0.04*	*0.53*	*0.22–1.30*	*0.17*
Time between conversion and CNI discontinuation	1.05	0.89–1.24	0.56			
No MMF at the time of TCMR or at month 3 Dose of MMF No steroids at the time of TCMR or at month 3 Dose of steroids	1.42 1.00 1.21 *1.14*	0.56–3.59 1–1.01 0.56–2.58 *1.02–1.28*	0.46 0.48 0.62 *0.02*	*1.1*	*0.96–1.26*	*0.17*

F, female subjects; ECD, extended criteria donor; KT, kidney transplantation; MMF, mycophenolate mofetil; eGFR, estimated glomerular filtration rate. Italic values: significative in univariate analysis. Bold values: significative in multivariate analysis.

Among KTRs with TCMR, 28/47 (59.6%) occurred in patients who were converted to belatacept during the first 6 months post transplantation (early conversion). We compared this population to the retrospective cohort in which 82 KTRs had early conversion to belatacept but no TCMR. In multivariate analysis (Table 4), lymphocyte count at the time of conversion and the dose of steroids used after the conversion were associated with the occurrence of TCMR after early belatacept conversion.

**TABLE 4:** Univariate and multivariate Cox analyses for determining factors associated with the occurrence of T cell-mediated rejection (TCMR) after early belatacept conversion (<6 months post KT).

	Univariate analysis			Multivariate analysis		
	HR	IC 95%	*p*	HR	IC 95%	*p*
Lymphocytes count at conversion	***1.00***	***1.00–1.01***	***0.003***	**1.01**	**1.00–1.01**	**0.003**
No tacrolimus before conversion	0.58	0.20–1.68	0.35			
Time between conversion and CNI discontinuation	1.10	0.88–1.39	0.40			
No MMF at the time of TCMR or at month 3 Dose of MMF No steroids at the time of TCMR or at month 3 Dose of steroids	1.63 1.00 0.54 ***1.24***	0.49–5.39 0.99–1.01 0.07–3.99 ***1.05–1.45***	0.42 0.96 0.58 ***0.009***	***1.15***	***1.03–1.41***	***0.01***

MMF, mycophenolate mofetil; eGFR, estimated glomerular filtration rate. Bold values: significative in multivariate analysis.

Among KTRs with TCMR 19/47 (40.4%) occurred in patients converted to belatacept after the first 6 months post-transplantation (late conversion). We compared this population to the retrospective cohort in which 156 KTRs were converted to belatacept late after transplantation but without TCMR. In multivariate analysis (Table 5), the absence of post-conversion use of steroids was associated with the occurrence of TCMR after belatacept late conversion.

**TABLE 5:** Univariate and multivariate Cox analyses for determining factors associated with the occurrence of T cell-mediated rejection (TCMR) after late belatacept conversion (>6 months post KT).

	Univariate analysis			Multivariate analysis		
	HR	IC 95%	*p*	HR	IC 95%	*p*
Lymphocytes count at conversion	1.00	0.99–1.01	0.77			
No tacrolimus before conversion	0.38	0.11–1.31	0.12			
Time between conversion and CNI discontinuation	1.01	0.75–1.37	0.94			
No MMF at the time of TCMR or at month 3 Dose of MMF No steroids at the time of TCMR or at month 3 Dose of steroids	*3.51* 1.00 ***3.43*** 1.14	*0.81–15.20* 0.99–1.01 ***1.39–8.44*** 0.69–1.11	*0.09* 0.96 ***0.007*** 0.28	*1.59* ***2.58***	*0.36–6.97* ***1.01–6.62***	*0.54* *0.04*

MMF, mycophenolate mofetil; eGFR, estimated glomerular filtration rate. Italic values: significative in univariate analysis. Bold values: significative in multivariate analysis.

### Rate of Opportunistic Infections (OPIs)

The rate of OPIs was not different between the 2 groups (*p* = 0.25). In the TCMR group: 8 KTRs (8/47: 17%) developed 9 episodes of OPI, all occurring after the diagnosis of TCMR: 4 cases of CMV disease, 3 cases of invasive aspergillosis, 1 case of varicella-zoster infection and 1 case of HHV8 associated Kaposi sarcoma. In the control group, 26 KTR (27/238: 10.9%) developed 33 episodes of OPI: 14 cases of CMV disease, 10 cases of pneumocystis pneumonia, 2 cases of JC Virus associated PML, 2 cases of EBV-associated PTLD, 2 cases of varicella-zoster infection, 1 case of tuberculosis, 1 case of toxoplasmosis and 1 case of aspergillosis.

## Discussion

This is the first report of the rate of kidney transplant rejection, both cellular and humoral, over a 10-year period in a large cohort of KTRs who were converted to belatacept as a rescue strategy. We confirm that the occurrence of acute rejection after conversion to belatacept appears to be less frequent than with *de novo* use. A major pitfall of the use of belatacept as a *de novo* strategy is the increased risk of TCMR compared to cyclosporine: in the BENEFIT study the rate of TCMR was 17%–24% at 1 year [1] and in the BENEFIT-EXT study, it was 18% at 1 year [17]. Nevertheless the occurrence of such rejection was not associated with worse graft survival or a poorer graft function at 8 years post KT. Regarding TCMR after conversion to belatacept in stable patients, the rate reported in the randomized study by Budde et al in a large cohort was 8% compared to 4% in the CNI arm [4]. When belatacept was used as a rescue strategy, the rate of TCMR was between 5.3% and 11.4% according to different retrospective studies [7, 8, 18] and was not significantly different between the belatacept and CNI arms in our retrospective study (4.3% in both arms) [5] and in the recently published study by Divard et al (4% in both arms) [10].

In contrast to the data from the original princeps study, in our study the risk of graft loss or deterioration of renal function after rejection was significant. Almost 50% of the rejections had V lesions. We observed 8 graft losses after rejection and death-censored graft survival was nearly 50% at 5 years post rejection. Some refractory allograft rejections to steroids justified being very cautious. Rejection occurred very early after the conversion, as in the *de novo* use and therefore very close biological follow-up has to be implemented after conversion to belatacept. Nevertheless after treatment of TCMR (mainly with steroids) 70% of the patients recovered an eGFR at least equivalent to that at the time of the conversion. We identified 2 factors associated with graft loss after TCMR: eGFR on the day of rejection and the discontinuation of belatacept after treatment. In patients with a good response to treatment of the rejection, we believe that belatacept should be continued in this context in patients with features of CNI toxicity before conversion. Moreover, we also reported 8 deaths after rejection, 7 of which were due to infectious causes. Clinicians have to be extremely cautious about the overall infectious risk in the follow-up of these patients with poor graft function presenting TCMR. We and other authors already reported on the risk of OPIs after belatacept conversion as a rescue strategy, mainly due to CMV disease and pneumocystis pneumonia [6, 19]. Prophylaxis against these 2 pathogens must be implemented, if not, after the treatment of rejection in this context. Nevertheless the rate of OPI was not different between the TCMR group and the control group but one striking feature is the occurrence and death from invasive aspergillosis in three patients in the TCMR group. Rejection is already known to be a risk factor for invasive aspergillosis [20] but there are no data on the specific impact of costimulation blockade in this context, except in lung transplant recipients [21].

Regarding factors associated with the occurrence of TCMR, the time from transplantation to conversion appears to be essential. We already suspected that the proportion of acute cellular rejection is probably higher in early conversion (<6 months) [18]. In early conversion, the factor associated with the occurrence of TCMR was the lymphocyte count. This could be explained by the global level of immunosuppression before the conversion in KTRs: the higher the lymphocyte count, the lower the level of immunosuppression and the higher the risk of TCMR after the switch. Attention should be paid to the CNI tapering regimen, CNI exposure, and maintenance of mycophenolic acid dosing during conversion to prevent rejection [22]. In patients with a high lymphocyte count a more progressive discontinuation of CNIs could be proposed, for example, if antithymocyte globulins are not used as an induction. Such a protocol has already been used in the *de novo* use of belatacept with a reduction in the rejection rate [23]. The use of mTOR inhibitors instead of mycophenolate mofetil could be another possibility [24]. The use of a more intensive regimen of belatacept does not reduce the rejection rate in the PRINCEPS study [1]. In late conversion, the absence of steroids after conversion was associated with the occurrence of TCMR after conversion in multivariate analysis. Nevertheless the rejection rate after 6 months is low and we do not believe that reintroducing steroids in all KTRs converted is indicated but could be discussed in patients close to transplantation (conversion 6–12 months post KT?). We need biomarkers to assess the real risk of rejection in patients treated with belatacept (CD86 occupancy [25, 26]? Belatacept Drug Monitoring [27] ? Immunomonitoring of T cells resistant to costimulation blockade? [12]). Monitoring donor-derived cell-free DNA [28] or urinary chemokines [29] could be helpful in this situation, but has never been tested following belatacept conversion.

One of the benefits of belatacept use is the low incidence of *de novo* DSA, both in *de novo* use [3] and in the conversion protocol [5]. Budde et al. reported in their published conversion randomized control trial that the rate of *de novo* DSA in the belatacept arm was 1% compared to 7% in the CNI arm [4]. We confirmed this point in the case of rescue conversion strategy (7.4% in the belatacept group versus 15/64%–23.4% in the CNI group; *P* = 0.01) [5]. This is the first report of the incidence of ABMR in a large cohort of KTRs converted to belatacept as a rescue strategy and this rate was very low (<1%). This result is in line with the BELACOR study [30] in which sensitized patients with preformed DSA (Mean Fluorescence Intensity 500–3,000) received *de novo* belatacept infusion and none of them developed ABMR.

The retrospective nature of the study raises the concern of substantial bias. Nevertheless the high number of TCMR cases reported in this multicenter cohort allows us to find factors associated with graft loss in this context and also factors associated with the occurrence of TCMR in both early and late switching. Moreover, a strength of our study is the homogeneous conversion protocol used in all included centers regarding the dose of belatacept and the decrease protocol of CNIs. Future randomized studies including this particular population of KTRs, with poor graft function are highly needed to accurately report the rejection rate in this context and to avoid potential bias.

In conclusion, we have reported for the first time a low incidence of both TCMR (5%) and ABMR (<1%) in a very large and significant cohort of KTRs who were converted to belatacept as a rescue strategy. We have shown that for patients with TCMR after conversion, high doses of steroids are effective, but in some patients rejection impacted both graft and patient survival. eGFR at the time of rejection and continuation of belatacept after treatment are determining factors for graft survival. We also demonstrated that early switching (<6 months) is a more risky situation for TCMR occurrence compared to late switching (>6 months) and that the level of immunosuppression is probably essential. New markers are highly needed to better identify patients at risk of TCMR post-conversion, in order to use this immunosuppressive drug with less fear.

## Data Availability

The raw data supporting the conclusions of this article will be made available by the authors, without undue reservation.

## Abbreviations

CNIs, calcineurin inhibitors; DSA, donor specific antibody; ECDs, extended criteria donors; eGFR, estimated glomerular filtration rate; IQR, interquartile range; KTRs, kidney transplant recipients; MDRD, Modification of Diet in Renal Disease formula; OPI, Opportunistic infections; PML, progressive multifocal leukoencephalopathy.

## References

[1] Vincenti F Rostaing L Grinyo J Rice K Steinberg S Gaite L Belatacept and Long-Term Outcomes in Kidney Transplantation. N Engl J Med (2016) 374(4):333–43. 10.1056/NEJMoa1506027 26816011

[2] Vanrenterghem Y Bresnahan B Campistol J Durrbach A Grinyó J Neumayer HH Belatacept-Based Regimens Are Associated With Improved Cardiovascular and Metabolic Risk Factors Compared With Cyclosporine in Kidney Transplant Recipients (BENEFIT and BENEFIT-EXT Studies). Transplantation (2011) 91(9):976–83. 10.1097/TP.0b013e31820c10eb 21372756

[3] Bray RA Gebel HM Townsend R Roberts ME Polinsky M Yang L De Novo Donor-Specific Antibodies in Belatacept-Treated vs Cyclosporine-Treated Kidney-Transplant Recipients: Post Hoc Analyses of the Randomized Phase III BENEFIT and BENEFIT-EXT Studies. Am J Transpl (2018) 18(7):1783–9. 10.1111/ajt.14721 PMC6055714 29509295

[4] Budde K Prashar R Haller H Rial MC Kamar N Agarwal A Conversion From Calcineurin Inhibitor to Belatacept-Based Maintenance Immunosuppression in Renal Transplant Recipients: A Randomized Phase 3b Trial. J Am Soc Nephrol (2021) 32:3252–64. 10.1681/ASN.2021050628 34706967 PMC8638403

[5] Bertrand D Matignon M Morel A Ludivine L Lemoine M Hanoy M Belatacept Rescue Conversion in Kidney Transplant Recipients With Vascular Lesions (Banff Cv Score >2): A Retrospective Cohort Study. Nephrol Dial Transpl (2023) 38(2):481–90. 10.1093/ndt/gfac178 35544123

[6] Bertrand D Chavarot N Gatault P Garrouste C Bouvier N Grall-Jezequel A Opportunistic Infections After Conversion to Belatacept in Kidney Transplantation. Nephrol Dial Transpl (2020) 35(2):336–45. 10.1093/ndt/gfz255 32030416

[7] Brakemeier S Kannenkeril D Dürr M Braun T Bachmann F Schmidt D Experience With Belatacept Rescue Therapy in Kidney Transplant Recipients. Transpl Int (2016) 29(11):1184–95. 10.1111/tri.12822 27514317

[8] Darres A Ulloa C Brakemeier S Garrouste C Bestard O Del Bello A Conversion to Belatacept in Maintenance Kidney Transplant Patients: A Retrospective Multicenter European Study. Transplantation (2018) 102(9):1545–52. 10.1097/TP.0000000000002192 29570163

[9] Morel A Hoisnard L Dudreuilh C Moktefi A Kheav D Pimentel A Three-Year Outcomes in Kidney Transplant Recipients Switched From Calcineurin Inhibitor-Based Regimens to Belatacept as a Rescue Therapy. Transpl Int (2022) 35:10228. 10.3389/ti.2022.10228 35497889 PMC9043102

[10] Divard G Aubert O Debiais-Deschamp C Raynaud M Goutaudier V Sablik M Long-Term Outcomes After Conversion to a Belatacept-Based Immunosuppression in Kidney Transplant Recipients. Clin J Am Soc Nephrol (2024) 19(5):628–37. 10.2215/CJN.0000000000000411 38265815 PMC11108246

[11] Cortes-Cerisuelo M Laurie SJ Mathews DV Winterberg PD Larsen CP Adams AB Increased Pretransplant Frequency of CD28+ CD4+ TEM Predicts Belatacept-Resistant Rejection in Human Renal Transplant Recipients. Am J Transpl (2017) 17(9):2350–62. 10.1111/ajt.14350 PMC5599135 28502091

[12] Mathews DV Wakwe WC Kim SC Lowe MC Breeden C Roberts ME Belatacept-Resistant Rejection Is Associated With CD28+ Memory CD8 T Cells. Am J Transpl (2017) 17(9):2285–99. 10.1111/ajt.14349 PMC5573634 28502128

[13] Loupy A Haas M Roufosse C Naesens M Adam B Afrouzian M The Banff 2019 Kidney Meeting Report (I): Updates on and Clarification of Criteria for T Cell- and Antibody-Mediated Rejection. Am J Transpl (2020) 20(9):2318–31. 10.1111/ajt.15898 PMC7496245 32463180

[14] Rostaing L Massari P Garcia VD Mancilla-Urrea E Nainan G del Carmen Rial M Switching From Calcineurin Inhibitor-Based Regimens to a Belatacept-Based Regimen in Renal Transplant Recipients: A Randomized Phase II Study. Clin J Am Soc Nephrol (2011) 6(2):430–9. 10.2215/CJN.05840710 21051752 PMC3052236

[15] Racusen LC Solez K Colvin RB Bonsib SM Castro MC Cavallo T The Banff 97 Working Classification of Renal Allograft Pathology. Kidney Int (1999) 55(2):713–23. 10.1046/j.1523-1755.1999.00299.x 9987096

[16] Fishman JA. Infection in Solid-Organ Transplant Recipients. N Engl J Med (2007) 357(25):2601–14. 10.1056/NEJMra064928 18094380

[17] Durrbach A Pestana JM Florman S Del Carmen Rial M Rostaing L Kuypers D Long-Term Outcomes in Belatacept- Versus Cyclosporine-Treated Recipients of Extended Criteria Donor Kidneys: Final Results From BENEFIT-EXT, a Phase III Randomized Study. Am J Transpl (2016) 16(11):3192–201. 10.1111/ajt.13830 PMC5516151 27130868

[18] Bertrand D Terrec F Etienne I Chavarot N Sberro R Gatault P Opportunistic Infections and Efficacy Following Conversion to Belatacept-Based Therapy After Kidney Transplantation: A French Multicenter Cohort. J Clin Med (2020) 9(11):3479. 10.3390/jcm9113479 33126667 PMC7693007

[19] Chavarot N Divard G Scemla A Amrouche L Aubert O Leruez-Ville M Increased Incidence and Unusual Presentations of CMV Disease in Kidney Transplant Recipients After Conversion to Belatacept. Am J Transpl (2020) 21:2448–58. 10.1111/ajt.16430 33283406

[20] Pérez-Jacoiste Asín MA López-Medrano F Fernández-Ruiz M Silva JT San Juan R Kontoyiannis DP Risk Factors for the Development of Invasive Aspergillosis After Kidney Transplantation: Systematic Review and Meta-Analysis. Am J Transpl (2021) 21(2):703–16. 10.1111/ajt.16248 32780498

[21] Bell E Pisano J Brown M Friedman D. An Unexpectedly High Incidence of Invasive Fungal Diseases in Solid Organ Transplant Recipients Taking Belatacept for Organ Rejection Prophylaxis: A Single-Center Retrospective Cohort Study. Open Forum Infect Dis (2024) 11(6):ofae158. 10.1093/ofid/ofae158 38887477 PMC11181179

[22] Yazdi M Kahwaji JM Meguerditchian S Lee R. Belatacept Conversion Protocols and Outcomes in Kidney Transplant Recipients. Transpl Proc (2021) 53(3):976–83. 10.1016/j.transproceed.2020.11.001 33478745

[23] Adams AB Goldstein J Garrett C Zhang R Patzer RE Newell KA Belatacept Combined With Transient Calcineurin Inhibitor Therapy Prevents Rejection and Promotes Improved Long-Term Renal Allograft Function. Am J Transpl (2017) 17(11):2922–36. 10.1111/ajt.14353 PMC5868947 28544101

[24] Ferguson R Grinyó J Vincenti F Kaufman DB Woodle ES Marder BA Immunosuppression With Belatacept-Based, Corticosteroid-Avoiding Regimens in *De Novo* Kidney Transplant Recipients. Am J Transpl (2011) 11(1):66–76. 10.1111/j.1600-6143.2010.03338.x 21114656

[25] de Graav GN Baan CC Clahsen-van Groningen MC Kraaijeveld R Dieterich M Verschoor W A Randomized Controlled Clinical Trial Comparing Belatacept With Tacrolimus After De Novo Kidney Transplantation. Transplantation (2017) 101(10):2571–81. 10.1097/TP.0000000000001755 28403127

[26] de Nattes T Lebourg L Etienne I Laurent C Lemoine M Dumont A CD86 Occupancy in Belatacept-Treated Kidney Transplant Patients Is Not Associated With Clinical and Infectious Outcomes. Am J Transpl (2022) 22(6):1691–8. 10.1111/ajt.17005 35181996

[27] Chhun S Trauchessec M Melicine S Nicolas F Miele A Lukic S A Validated LC-MS/MS Method for Performing Belatacept Drug Monitoring in Renal Transplantation. Biomedicines (2023) 11(11):2955. 10.3390/biomedicines11112955 38001955 PMC10669563

[28] Aubert O Ursule-Dufait C Brousse R Gueguen J Racapé M Raynaud M Cell-Free DNA for the Detection of Kidney Allograft Rejection. Nat Med (2024) 30(8):2320–7. 10.1038/s41591-024-03087-3 38824959 PMC11333280

[29] Tinel C Sauvaget V Aouni L Lamarthée B Terzi F Legendre C Transforming Kidney Transplant Monitoring With Urine CXCL9 and CXCL10: Practical Clinical Implementation. Sci Rep (2024) 14(1):20357. 10.1038/s41598-024-70390-x 39223175 PMC11369285

[30] Leibler C Matignon M Moktefi A Samson C Zarour A Malard S Belatacept in Renal Transplant Recipient With Mild Immunologic Risk Factor: A Pilot Prospective Study (BELACOR). Am J Transpl (2019) 19(3):894–906. 10.1111/ajt.15229 30582270

